# COL^R^ *Acinetobacter baumannii* sRNA Signatures: Computational Comparative Identification and Biological Targets

**DOI:** 10.3389/fmicb.2019.03075

**Published:** 2020-01-17

**Authors:** Viviana Cafiso, Stefano Stracquadanio, Flavia Lo Verde, Veronica Dovere, Alessandra Zega, Giuseppe Pigola, Jesús Aranda, Stefania Stefani

**Affiliations:** ^1^Department of Biomedical and Biotechnological Sciences, University of Catania, Catania, Italy; ^2^Department of Translational Research and New Technology in Medicine and Surgery, Azienda Ospedaliero Universitaria Pisana, University of Pisa, Pisa, Italy; ^3^Department of Clinical and Experimental Medicine, University of Catania, Catania, Italy; ^4^Departament de Genètica i Microbiologia, Facultat de Biociènces, Universitat Autònoma de Barcelona, Barcelona, Spain

**Keywords:** XDR *Acinetobacter baumannii*, colistin resistance, small RNAs, Illumina RNA-seq, bioinformatics

## Abstract

Multidrug-Resistant (MDR) and Extensively Drug Resistant (XDR) *Acinetobacter baumannii* (*Ab*) represent a serious cause of healthcare-associated infections worldwide. Currently, the available treatment options are very restricted and colistin-based therapies are last-line treatments of these infections, even though colistin resistant (COL^R^) *Ab* have rarely been isolated yet. In bacteria, small non-coding RNAs (sRNAs) have been implicated in regulatory pathways of different biological functions, however, no knowledge exists about the sRNA role on the biological adaptation in COL^R^ *Ab*. Our study investigated two Italian XDR isogenic colistin-susceptible/resistant (COL^S/R^) *Ab* strain-pairs to discover new sRNA signatures. Comparative sRNA transcriptome (sRNAome) analyses were carried out by Illumina RNA-seq using both a Tru-Seq and a Short Insert library, whilst *Ab* ATCC 17978 and ACICU Reference Genome assembly, mapping, annotation and statistically significant differential expression (*q*-value ≤ 0.01) of the raw reads were performed by the Rockhopper tool. A computational filtering, sorting only similarly statistically significant differentially expressed (DE) sRNAs mapping on the same gene in both COL^R^ *Ab* isolates was conducted. COL^R^ vs. COL^S^ sRNAome, analyzed integrating the DE sRNAs obtained from the two different libraries, revealed some statistically significant DE sRNAs in COL^R^ *Ab*. In detail, we found: (i) two different under-expressed *cis*-acting sRNAs (*Ab*sRNA_1_ and *Ab*sRNA_2_) mapping in antisense orientation the 16S rRNA gene A1S_r01, (ii) one under-expressed *cis*-acting sRNA (*Ab*sRNA_3_) targeting the A1S_2505 gene (hypothetical protein), (iii) one under-expressed microRNA-size small RNA fragment (*Ab*sRNA_4_) and its pre-micro*Ab*sRNA_4_ targeting the A1S_0501 gene (hypothetical protein), (iv) as well as an over-expressed microRNA-size small RNA fragment (*Ab*sRNA_5_) and its pre-micro*Ab*sRNA_5_ targeting the A1S_3097 gene (signal peptide). Custom TaqMan^®^ probe-based real-time qPCRs validated the expression pattern of the selected sRNA candidates shown by RNA-seq. Furthermore, analysis on sRNA ΔA1S_r01, ΔA1S_2505 as well as the over-expressed A1S_3097 mutants revealed no effects on colistin resistance. Our study, for the first time, found the sRNAome signatures of clinical COL^R^ *Ab* with a computational prediction of their targets related to protein synthesis, host-microbe interaction and other different biological functions, including biofilm production, cell-cycle control, virulence, and antibiotic-resistance.

## Introduction

The Multidrug-Resistant (MDR) Gram-negative pathogens included recently in the WHO black list (Tacconelli et al., 2018), i.e., *Acinetobacter, Pseudomonas, and various Enterobacterales* (including *Klebsiella, E. coli, Serratia, and Proteus*) represent a serious health problem worldwide. In particular, focusing on the *Acinetobacter baumannii* infections, the therapeutic options for their treatment are very limited and mainly draw on colistin-based therapies (Hancock and Chapple, 1999; Zavascki et al., 2007; Vila and Pachón, 2012). Therefore, the increasing use of colistin (COL) and the spread of colistin resistant (COL^R^) can consequently determine an increase in polymyxin resistance onset (Ko et al., 2007; Gales et al., 2011).

Small non-coding RNAs (sRNAs) [∼30–300 nucleotides (nt) length] have been recognized as a major class of regulatory molecules in bacteria (Barquist and Vogel, 2015). They unroll a regulatory function affecting gene expression – by base pairing to their related target mRNA – modulating transcription, translation, mRNA stability, DNA maintenance, and silencing. Functionally, sRNAs are involved in the regulation of a wide range of physiological responses, reacting to environmental signals, such as pH or temperature shifts (Wassarman, 2002). They can help the modulation of changes in cellular metabolism to optimize use of available nutrients and improve the probability for survival, as well as contributing to virulence (Chabelskaya et al., 2010; Gottesman and Storz, 2011; Sayed et al., 2012; Álvarez-Fraga et al., 2017; Kröger et al., 2018).

sRNAs act in *trans* or *cis* depending on the transcription start position where the sRNA is transcribed with respect to their regulated gene. *Trans-*encoded sRNAs are transcribed at a genetic locus separated from the gene that they regulate and they often work via an imperfect base pairing with their target mRNAs. Conversely, *cis*-acting sRNAs are transcribed from the same genetic locus that they regulate even thought in an antisense orientation to the target gene. Since transcribed proximally to their target, *cis*-antisense RNAs share a perfect match with their targets allowing duplex formation and a stringent regulation (Thomason and Storz, 2010; Chang et al., 2015; Georg and Hess, 2018).

A third class of small RNA, the microRNA-size small RNA fragments (∼15–26 nt) have been identified in a few different species of bacteria by next generation sequencing (NGS). These molecules could originate from a cut of a longer precursor (pre-sRNA) in analogy with maturation of the eukaryotic microRNAs (Bloch et al., 2017).

Few mechanisms have been proposed to the underlying colistin resistance in *Ab* (Li et al., 2006; Adams et al., 2009; Falagas et al., 2010; Moffatt et al., 2010; Arroyo et al., 2011; Cai et al., 2012; Hood et al., 2013; Parra-Millán et al., 2018; Cafiso et al., 2019), however, no investigations have been conducted so far on the role that sRNAs exert on the biological adaptations of colistin resistance acquisition. Few studies have analyzed the sRNA contribution to the *A. baumannii* biology and antimicrobial resistance. Weiss et al. (2016) offer a detailed view of the sRNA content of *Ab* and provide new insights into the evolution and role of these regulatory molecules. In detail, using RNA-seq, 78 *Ab* sRNAs were identified in the AB5075 background, grouped in six classes of similar sRNAs, with one particularly abundant and homologous to regulatory C4 antisense RNAs found in bacteriophages. Sharma et al. (2014) found three new sRNAs, namely AbsR11, AbsR25, and AbsR28, hypothesizing an sRNA involvement in the regulation of antibiotic resistance in bacteria, specifically in cryptic *A. baumannii*.

Our study aimed to gain new insight on the sRNA signature and biological target of clinical COL^R^ *Ab* by high-throughput technology RNA-seq.

Our data define the distinctive signatures of COL^R^ *Ab* sRNAs revealing computational predicted targets involved in the protein synthesis machinery, host-microbe interactions, pathways involved in biofilm production, cell cycle control, virulence, and antibiotic-resistance.

## Materials and Methods

### Bacterial Strains

Two Italian Extensively Drug Resistant (XDR) isogenic colistin-susceptible/resistant (COL^S/R^) clinical *Ab* strain-pairs (1-S/R, 2-S/R) were investigated. *Ab* strain-pair source, antimicrobial susceptibility, molecular characterization, genomic epidemiology, and phylogeny were previously characterized (Cafiso et al., 2019).

### RNA-Seq

To optimize data, RNA-seq was carried out using the Illumina Mi-seq Standard pipeline on two biological replicates consisting of two different libraries of each strain i.e., a Single-end library with 50 bp reads (Short-Insert library) and a Paired-end Read library with 150 bp reads (Tru-Seq library).

For RNA extraction, a single colony of each *Ab* strain was grown in 10 ml of Cation-adjusted Mueller-Hinton broth (Ca-MHB) (Becton Dickinson) and incubated at 37°C overnight. The overnight cultures were then diluted 1:50 in 50 ml of Ca-MHB in a sterile 250 ml flask and incubated with shaking at 250 rpm at 37°C. Bacterial pellets were harvested at mid-log growth phase (OD_600_ 0.6 ∼1.5 × 10^7^ CFU/ml, 18 h) according to the strain growth-curves (Supplementary Figure S1).

RNA extraction was performed using the NucleoSpin RNA kit (Macherey-Nagel, Düren, Germany) following the manufacturer’s protocol with minor modifications according to the previously published protocols (Cafiso et al., 2019).

The total RNA quality was checked by the 2200 TapeStation RNA Screen Tape device (Agilent, Santa Clara, CA, United States) and the RNA concentrations were determined using an ND-1000 spectrophotometer (NanoDrop, Wilmington, DE, United States). RNA Integrity Number (RIN) values, ranging from 1 to 10 with 10 being the highest quality, was determined by the Agilent TapeStation 2200 system. Only RNAs with preserved 16S and 23S peaks and with RIN values >8 were used for library construction. The RIN values >8 indicated intact and high quality RNA samples usable for downstream applications as previously published (Fleige and Pfaffl, 2006).

### Library Preparation and Sequencing

The Tru-Seq library (TS) was an A Paired-end library with reads of 150 bp and average insert size of 350/400 bp. After sequencing, raw reads were processed using FastQC v0.11.2 to check data quality and, then, reads were trimmed by Trimmomatic v.0.33.2 to remove the adapters for Paired-end reads. A minimum base quality of 15 over a 4-bases sliding-window was required. Only trimmed reads with a length above 36 nucleotides were included in the downstream analysis (Cafiso et al., 2019).

The Short-Insert library (SI) was processed with an A Single-end stranded library with reads of 50 bp. After sequencing, raw reads were processed using FastQC v0.11.2 to evaluate data quality. Reads were then trimmed using Trimmomatic v.0.33.2 to remove sequencing adapters for Single-end reads, requiring a minimum base quality of 15 (Phred scale) and a minimum read length of 15 nucleotides. Only trimmed reads were included in the downstream analysis (Cafiso et al., 2019).

### Tru-Seq and Short-Insert Library Analysis

TS and SI RNA-seq reads were aligned on *Ab* ATCC 17978 (CP000521.1) and on *Ab* ACICU (CP000863.1) Reference Genomes, assembled and quantified using Rockhopper v.2.03 opportunely developed to investigate the bacterial gene structures and transcriptomes as well as validated to identify novel sRNAs (McClure et al., 2013; Tjaden, 2015; Cafiso et al., 2019).

To obtain the data, analyses were carried out with default parameters and verbose output. Rockhopper normalizes read counts for each sample using the upper quartile gene expression level. Starting from the *p*-values calculated according to the Anders and Huber approach, differentially expressed genes (DEGs) were selected as statistically significant by computing *q*-values ≤ 0.01 based on the Benjamini–Hochberg correction with a false discovery rate of <1%. In addition, Rockhopper is a tool using biological replicates when available and surrogates when biological replicates for two different conditions are unavailable, considering the two conditions under investigation surrogate replicates for each other (McClure et al., 2013).

### Comparative sRNA Prediction

A double computational filtering was carried out on the library analysis data outputs, first for the DE sRNAs in the COL^R^ strains versus their COL^S^ counterparts and, thus, to identify only those mapping on the same gene in both COL^R^ *Ab* isolates with a statistically significant *q*-value ≤ 0.01.

### Determination of Small RNA Functional Categories

Functional categories of small RNA target genes were investigated by different bioinformatic tools including BLAST, PANTHER (Protein ANalysis THrough Evolutionary Relationships) Classification System, Gene Ontology (GO) Consortium, ExPASy, and KEGG.

### RNA-Seq Data Accession Number

RNA-seq data of the two different libraries have been deposited in the NCBI GEO database under study accession no. GSE109951.

### I-Tasser *ab initio* Structure Modeling

For the I-Tasser (Iterative Threading asseMBLY Refinement) analysis (Zhang, 2009; Roy et al., 2010, 2012; Yang and Zhang, 2015), the first N-terminal 120 AA of the *Ab* ATCC 17978 *Ab*sRNA_2_ target (A1S_2505) was selected as a target to obtain more significant predictions, as the C-terminal part of the A1S_2505 protein was excluded because of its low coverage with threading templates identified from the PDB-library: MTYQYHDESIVTELPEDTVFVFGSNMAGQHGSGAARVASQ HFGAVEGVGRGWAGQSFAIPTLNEHIQQMPLSQIEHYVEDF KVYAKNHPKMKYFVTALGCGIAGYK*VS*EIAPLFKGIHHN.

For the analysis of the micro*Ab*sRNA_4_ target (A1S_0501) the whole FASTA sequence of the protein was used as a template.

### Validation of sRNA-Seq Expression

*Ab*sRNA_3_ and micro*Ab*sRNA_5_ were selected to be validated since they are representative of under-expression and over-expression, respectively. Particularly, their RNA-seq expression levels were validated by Custom TaqMan^®^ Small RNA Assays as follow: a single colony of each strain was grown in 10 ml of Ca-MHB (Becton Dickinson) and incubated at 37°C overnight. The overnight cultures were diluted 1:50 in 50 ml of Ca-MHB in a sterile 250 ml flask and incubated with shaking at 250 rpm under normal atmospheric conditions at 37°C. Bacterial pellets were harvested in mid-log growth phase, lysed by adding lysozyme (10 mg/ml) and incubated for 1 h at 37°C. Small RNA extraction was performed by using the *mir*Vana^TM^ miRNA Isolation Kit (Ambion, Austin, TX, United States) according to the manufacture’s protocol following the enrichment procedure for small RNAs. Extracted sRNAs were quantified using Eppendorf BioPhotometer D30 to assess their quality and to properly dilute them to the amount suggested by Custom TaqMan^®^ Small RNA Assays protocol (Applied Biosystems^TM^), then a stem-loop qRT-PCR was performed, one of the most commonly used real-time PCR approaches to quantify sRNAs. The quantification assay was divided into two steps: i) RNA was reverse-transcribed into cDNA using a stem-loop specific primer, and ii) the quantified RT product was consequently used as a template for real-time qPCR with TaqMan^®^ Fast Advanced Master Mix (Applied Biosystems). The stem-loop specific primer used for RT and the specific TaqMan^®^ probes and primers used for real-time qPCR were provided by Custom TaqMan^®^ Small RNA Assay Design Tool on the Applied Biosystems website^TM^. All real-time qPCRs were performed in triplicate, using Agilent AriaMx Real-Time PCR System, with three different biological replicates using one of the thermal profiles suggested by the Custom TaqMan^®^ Small RNA Assays protocol, which provided a first enzymatic activation step of 95°C for 10 min, followed by 40 cycles of 95°C for 15 s and 60°C for 60 s (Salone and Rederstorff, 2015). The expression levels of *Ab*sRNA_3_ and micro*Ab*sRNA_5_ are shown as the increment/decrement fold-change (FC) in COL^R^ (1-R, 2-R) vs. COL^S^ strains (1-S, 2-S) in RNA-seq and real-time qPCR.

### Statistics

*Ab*sRNA_3_ and micro*Ab*sRNA_5_ expression levels found by Custom TaqMan^®^ probe-based real-time qPCRs were expressed as means ± standard deviations and analyzed by the one-way analysis of variance (ANOVA) using the on-line Free Statistics Calculators-DanielSoper (Soper, 2019) considering a *p*-value ≤ 0.01 as statistically significant.

### *Ab* ATCC 17978 Mutant Construction

Due to the difficult to manipulate the clinical *A. baumannii* strain-pairs related to the lack of antibiotic markers for the mutants selection, *A. baumannii* ATCC 17978 strain was used to generate ΔA1S_r01 and ΔA1S_2505 mutants as well as to overexpress the A1S_3097 gene.

Gene inactivation was carried out as previously described (Aranda et al., 2010). Briefly, an internal fragment of the target gene was PCR-amplified from the *A. baumannii* ATCC 17978 genome, using the appropriated primers (listed in Table 1). The internal fragment was cloned into the pCR-BluntII-TOPO plasmid (Invitrogen), introduced by electroporation in *Escherichia coli* DH5α (Clontech) and selected in kanamycin-containing LB plates. Purified plasmids were then introduced by electroporation in *A. baumannii* ATCC 17978 strain and selected on kanamycin-containing plates. Recombinant clones were confirmed by sequencing (Macrogen) of the PCR products obtained by using the appropriate primers (Table 1). For overexpression, the A1S_3097 gene was cloned, using the indicated primers (Table 1), into *Xba*I-*Nco*I sites of the pET-RA vector (Aranda et al., 2010). The recombinant plasmid was introduced in *E. coli* DH5α and once correct construction was verified by both PCR and sequencing (Macrogen), in ATCC 17978. Finally, *A. baumannii* transformants overexpressing the A1S_3097 gene were selected on rifampicin- and kanamycin-containing plates and confirmed by PCR with the pETRAFW and pETRARV primers (Table 1).

**TABLE 1:** Oligonucleotides used in this work.

**Name**	**Sequence (5**′ **to 3**′**)**	**Application**
A1S_r01intF	GTAGCTTGCTACTGGACC	Construction of the ΔA1S_r01 mutant
A1S_r01intR	AGTAAATCCGATTAACGC	Construction of the ΔA1S_r01 mutant
A1S_r01extF	CTTAACACATGCAAGTCG	Confirmation of the ΔA1S_r01 mutant
A1S_r01extR	ATAAGCCGCCTACGCACG	Confirmation of the ΔA1S_r01 mutant
A1S_2505intF	GCACGTGTTGCCAGTCAG	Construction of the ΔA1S_2505 mutant
A1S_2505intR	TCACTGCCGTGATTGAAG	Construction of the ΔA1S_2505 mutant
A1S_2505extF	ATGGCTGGACAACATGGTAG	Confirmation of the ΔA1S_2505 mutant
A1S_2505extR	TTAACGGTTAATAGGGTG	Confirmation of the ΔA1S_2505 mutant
A1S_3097FXbaI	ATCGTCTAGAATGGGTGTTGTTGCTGATAG	Overexpression of the A1S_3097 gene
A1S_3097RNcoI	ATCGCCATGGTTATGGTTGAACGTCGGC	Overexpression of the A1S_3097 gene
pETRAFW	TTCTTCGTGAAATAGTGATGATTTTT	Sequencing primer for pET-RA plasmid
pETRARV	CTGTTTCATATGATCTGGGTATC	Sequencing primer for pET-RA plasmid
M13FpUC	GTTTTCCCAGTCACGAC	Sequencing primer for the pCR-BluntII-TOPO plasmid
M13RpUC	CAGGAAACAGCTATGAC	Sequencing primer for the pCR-BluntII-TOPO plasmid

## Results

### Comparative Transcriptome Analysis

To define the characterizing sRNA traits of the two COL^R^ vs. COL^S^ clinical *Ab* strains, a comparative analysis of the DE sRNAs was conducted by a computational double cross-filtering.

RNA-sequencing generated 1,307,792 – 1,175,327 – 1,173,332 – 1,270,020 total reads in 1-S, 1-R, 2-S, and 2-R, respectively, with 96, 96, 72, and 93% mapped reads on *Ab* ATCC 17978; as well as 97, 97, 76, and 95% reads aligned on *Ab* ACICU for the TS library, whilst 2,353,045 – 2,041,858 – 1,804,167 – 1,819,349 total reads in 1-S, 1-R, 2-S, and 2-R, respectively, with 57, 56, 53, and 56% mapped reads on *Ab* ATCC 17978 and 59, 58, 54, and 57% on *Ab* ACICU for the SI library. RNome structures were previously published (Cafiso et al., 2019).

As shown in Tables 2, 3 and Supplementary Table S1, the comparative statistically significant filtering-analysis of the sRNAome sorting for DE sRNAs of the COL^R^ vs. COL^S^ *Ab* parental strains returned two different under-expressed *cis*-acting sRNAs (*Ab*sRNA_1_ and *Ab*sRNA_2_) mapping in antisense orientation the 16S rRNA gene A1S_r01, one under-expressed *cis*-acting sRNA (*Ab*sRNA_3_) targeting the A1S_ 2505/ACICU_02783 gene (hypothetical protein), one under-expressed microRNA-size small RNA fragment (*Ab*sRNA_4_) and its pre-micro*Ab*sRNA_4_ targeting the A1S_0501 gene (hypothetical protein), as well as an over-expressed microRNA-size small RNA fragment (*Ab*sRNA_5_) and its pre-micro*Ab*sRNA_5_ targeting the A1S_3097 gene coding a signal peptide involved in the cytokinin biosynthesis. In detail, the two different *cis*-acting sRNAs – mapping on the *Ab* ATCC 17978 Reference Genome – targeted two different positions (Table 2) of the A1S_r01 gene with a size of 35 bp in strain-pair 1 (*Ab*sRNA_1_) and 39 bp in strain-pair 2 (*Ab*sRNA_2_), no mapped regions were found on the *Ab* ACICU Reference Genome. The 70–75 nt *cis*-acting *Ab*sRNA_3_ mapped in an analog position both on *Ab* ATCC 17978 and *Ab* ACICU Reference Genomes targeting the A1S_2505/ACICU_02783 gene in both the *Ab* strain-pairs. The 107 nt pre-micro*Ab*sRNA_4_ covering the A1S_0501 mapping on *Ab* ATCC 17978 Reference Genome and its smaller fragment of 21 bp (micro*Ab*sRNA_4_) were found in *Ab* strain-pair 2 and in *Ab* strain-pair 1, respectively. Similarly, a 228 pre-micro*Ab*sRNA_5_ targeting the A1S_3097 gene in *Ab* strain-pair 2 and its inner fragment of 20 nt in *Ab* strain-pair 1 were found. Furthermore, both *Ab* strain-pairs presented the same aforementioned sRNAs with similar expression profiles (over- or under-expression), though the *q*-value did not allow, in some cases, to return the same fragments in both strain-pairs, considering the two different genomic annotations. Moreover, the only sRNA with a statistically significant expression in *Ab* ACICU Reference Genome was ACICU_02783 (*Ab*sRNA_3_) in both strain-pairs (Supplementary Table S1).

**TABLE 2:** sRNA nucleotide positions in *Ab* ATCC 17978 reference genome.

**sRNA interaction mechanisms**	***Ab*^a^ ATCC 17978 Locus Tag**	**Strain-pair 1**					**Strain-pair 2**				
		**sRNA**	**RefGen^∗^ position (nt)**		**sRNA size (bp)**	**Library^b^**	**sRNA**	**RefGen^∗^ position (nt)**		**sRNA size (bp)**	**Library^b^**
*cis-*acting	A1S_r01	*Ab*sRNA_1_	194580	194546	35	SI	*Ab*sRNA_2_	194033	193995	39	SI
*cis-*acting	A1S_2505	*Ab*sRNA_3_	2904500	2904426	75	SI	*Ab*sRNA_3_	2904495	2904426	70	SI
microRNA-size small RNA fragment	A1S_0501	micro*Ab*sRNA_4_	545842	545822	21	SI	pre-micro*Ab*sRNA_4_	546023	545917	107	TS
microRNA-size small RNA fragment	A1S_3097	micro*Ab*sRNA_5_	3577445	3577464	20	SI	pre-micro*Ab*sRNA_5_	3577336	3577563	228	SI

*^*a*^*Ab, Acinetobacter baumannii*; ^*b*^SI, Short-Insert Library; TS, Tru-Seq Library; ^∗^RefGen, Reference Genome.*

**TABLE 3:** Comparative sRNAs of the *A. baumannii* strains.

***Ab*sRNA**	***Ab*^a^ ATCC 17978 Locus Tag**	**Description**	**I-Tasser *Ab Initio* modeling prediction**	**COG^b^**	**GO number^c^**	**RPKM^d^ of RNA-seq data annotated on *Ab* ATCC 17978^a^*q*-value ≤ 0.01^∙^**				**Expression profile**
						**1-R**	**1-S**	**2-R**	**2-S**	
*Ab*sRNA_1_ *Ab*sRNA_2_	A1S_r01	Antisense sRNA: 16S ribosomal RNA	–	–	–	105	523	19	780	↓
*Ab*sRNA_3_	A1S_2505	Antisense sRNA: hypothetical protein	O-Acyl-ADP-ribose deacylase	–	–	187	647	6	352	↓
micro*Ab*sRNA_4_ pre-micro*Ab*sRNA_4_	A1S_0501	Antisense sRNA: hypothetical protein	Bacterial actin MreB assembles in complex with cell shape protein RodZ	1426	GO:0016021	111	696	5	310	↓
micro*Ab*sRNA_5_ pre-micro*Ab*sRNA_5_	A1S_3097	Antisense sRNA: signal peptide for cytokinin biosynthesis	–	R	GO:0009691	173	0	472	15	↑

*^*a*^*Ab, Acinetobacter baumannii*; ^*b*^R, general functional prediction only; 1426, Cytoskeletal protein RodZ, contains Xre-like HTH and DUF4115 domains; ^*c*^GO numbers refer only to the biological process; ^*d*^RPKM, Reads per kilo base per million mapped reads; **^∙^***q*-value ≤ 0.01 according to the Rockhopper guidelines were considered statistically significant.*

The sRNA nucleotide positions (transcription start and stop) reported by Rockhopper tool were shown in Table 2. In addition, none of these sRNAs targeted the 5′ or 3′ untranslated regions (UTR) of their target genes.

### I-Tasser

To predict the putative role of *Ab*sRNA_2_, an A1S_2505/ACICU_02783 conserved domain (CD) BLAST search and I-Tasser *ab initio* protein structure prediction were computationally investigated. The three-dimensional structure of a protein can be very informative and useful to understand functional characteristics of proteins with unknown functions. This is because the structure of a protein provides the precise molecular details that often facilitate experimental characterization of an expected function. In a case in which there is no expected function, the structure of a protein can be used to facilitate its functional predictions by using the structure as a search template for better-characterized proteins that share regions of structural similarity (Kemege et al., 2011). The CD-BLAST search provided a match with the PHA00684 super family (cl10259) domain related to a protein of unknown function. On the contrary, analyzing the concordances of the highest significant prediction of the I-Tasser TM-align structural alignment and the COACH Predicted biological function, we resolved the structure and biological function as similar to the Orphan Macrodomain Protein (human C6orf130) with O-Acyl-ADP-ribose deacylase activity. In particular, the closest structural similarity of the targeted A1S_2505 was the PDB-Hit 2lgrA (TM-score 0.925) matching the human protein C6orf130, previously published as an Orphan Macrodomain Protein (human C6orf130) with an O-Acyl-ADP-ribose deacylase activity, which catalyzes the deacylation of O-acetyl-ADP-ribose, O-propionyl-ADP-ribose, and O-butyryl-ADP-ribose to produce ADP-ribose (ADP-r) with acetate, propionate, and butyrate, respectively. Due to the structural similarity, we can speculate that A1S_2505/ACICU_02783 could have a function similar to O-Acyl-ADP-ribose deacylase. This structural prediction was also supported by the COACH Predicted biological function, 2l8rA, defining the human protein C6orf130 in complex with ADP-ribose (C-score 0.59), matching the O-acetyl-ADP-ribose deacetylase receptor binding the ADP-ribose in the AA residues – G19 D20 L21 F22 H32 C33 I34 S35 R39 A42 I44 A45 L47 A87 P118 R119 I120 G121 C122 G123 L124 D125 Y150 L152- representing the binding sites. This ligand binding site (BL0101984) showed GO Molecular Functions of purine nucleoside binding (GO:0001883), hydrolase activity (GO:0016787) and deacetylase activity (GO:0019213) as well as the GO Biological Process of the purine nucleoside metabolic process (GO:0042278). Regarding the computational prediction of the putative role of *Ab*sRNA_3_, the A1S_0501 hypothetical protein referred to an integral membrane component (GO:0016021), whilst the CD BLAST search provided a CD of the cytoskeletal protein RodZ containing Xre-like HTH and DUF4115 domains related to the cell-cycle control, cell division, and chromosome partitioning (cl34261 Superfamily). This result was also supported by the I-Tasser predictions. By LOMETS, the A1S_0501 protein showed homology with the highest Norm *Z*-score (1.71) and 0.33 coverage with 2wus hit referred as a bacterial structural protein actin MreB that can be complexed with the cell shape protein RodZ. As regards the A1S_0501 GO and the consensus prediction of GO term, obtained from I-Tasser, the A1S_0501 protein showed a molecular function of DNA polymerase activity (GO:0034061) (GO-score 0.38) and DNA binding (GO:0003677) (GO-score 0.34) and the biological process of nucleic acid metabolism (GO:0090304) (GO-score 0.38).

### Validation of sRNA Expression Profile

**FIGURE 1:**
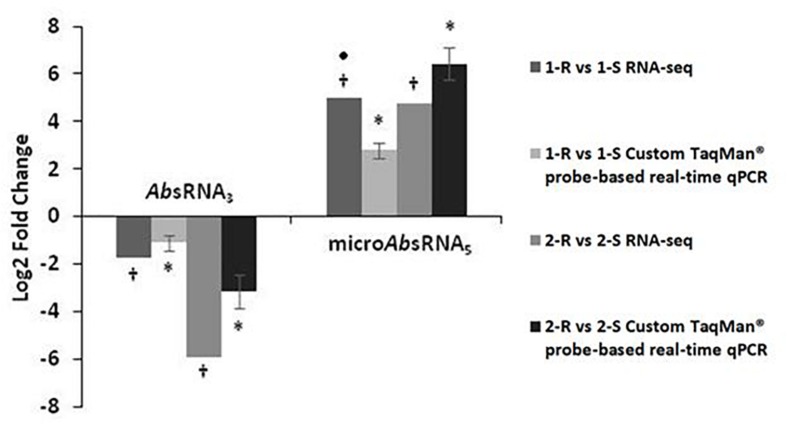
Custom TaqMan probe-based real-time qPCR validation of RNA-seq expression data on colistin resistant (COL^R^) characterizing sRNAs.a value of 5-Fold Changes (FC) was indicated for incalculable FC due to the presence of a 0 value in one of the strains. ^∗^*p*-value ≤ 0.01 obtained by a Student’s *T*-test and †*q*-value ≤ 0.01 according to the Rockhopper guidelines were considered statistically significant.

Custom TaqMan^®^ probe-based real-time qPCRs, dedicated for the analysis of bacterial sRNAs, validated and confirmed the RNA-seq expression profiles of two selected sRNA candidates: *Ab*sRNA_3_ and micro*Ab*sRNA_5_ in both *Ab* strain-pairs, as shown in Figure 1. In detail, *Ab*sRNA_3_ had a statistically significant under-expression (*p*-value ≤ 0.01), whilst micro*Ab*sRNA_5_ showed a statistically significant over-expression (*p*-value ≤ 0.01) in both COL^R^ *Ab* strains compared with their COL^S^ parents.

### sRNA Target Mutants

No COL MIC changes with respect to the wild-type *Ab* ATCC 17978 (COL-MIC 1 mg/L) were observed in the ΔA1S_r01 *Ab* ATCC 17978 and ΔA1S_2505 *Ab* ATCC 17978 mutants as well as in the WT + A1S_3097 (Table 4).

**TABLE 4:** COL MIC in *Ab* ATCC 17978 mutants.

**Phenotype of wild type and derivative strains**	**Colistin MIC (mg/L)**
Wild type (WT) *Ab* ATCC 17978	1
ΔA1S_r01	1
ΔA1S_2505	1
WT + clinical strain derived A1S_3097	1

## Discussion

The comparative (sRNAome) integrated to bioinformatics, computational cross-double filtering and experimental validations of COL^R^ versus COL^S^ *Ab* strain-pair revealed distinctive small RNA signatures in COL^R^ *Ab*.

Small non-coding RNAs have been identified so far as crucial regulatory elements in bacteria showing high structural diversity and molecular action mechanisms. The most intensively studied prokaryotic sRNA regulators are *cis*-acting sRNAs or *trans*-encoded sRNAs, however, the more recent discovery of microRNAs in prokaryotes represents a challenging field of investigation regarding bacterial regulatory mechanisms.

Our clinical COL^R^ *Ab* were distinguished by 3 *cis*-acting sRNAs and 2 microRNA-size small RNA fragments involved in different biological networks. These distinctive features span a different area of bacterial biology involving protein synthesis apparatus, host-microbe interactions, biofilm production, cell-cycle control, virulence and antibiotic resistance that emerge as sRNA targets. Notably, they do not seem apparently related to colistin resistance mechanism as demonstrated by our preliminary data on ΔA1S_r01, ΔA1S_2505 and high expressed A1S_3097 *Ab* ATCC 17978 mutants showing no COL MIC variations of mutants compared to the WT *Ab* ATCC 17978 – reflecting, however, the wide biological adaptations that the co-existence of colistin resistance and the XDR profile implies.

Regarding the novel *Ab*sRNA_1_, *Ab*sRNA_2_ and *Ab*sRNA_3_, we can speculatively assume that via a *cis*-antisense regulation mechanism they could post-transcriptionally regulate the target translation. On the contrary, no regulation appears at the transcriptional level as demonstrated by the lack of statistically significant differential expression in these *Ab*sRNA targets according to previously published transcriptomic data (Cafiso et al., 2019). On top, in COL^R^ strains, *Ab*sRNA_1_ and *Ab*sRNA_2_ under-expression could indicate that they are characterized by sRNAs modulating the protein synthesis machinery and the amount of active ribosomes in agreement with other previous findings (Fernández-Reyes et al., 2009). The occurrence of the under-expressed *Ab*sRNA_3_ – targeting the O-acetyl-ADP-ribose – provided evidence that COL^R^ *Ab* are distinguished by an *Ab*sRNA_3_ involved in the RNase III inhibition previously associated with different biological functions, including biofilm production, virulence, and antibiotic resistance in Gram-negative bacteria (Chen et al., 2011; Kim et al., 2013; Song et al., 2014). In fact, the O-acetyl-ADP-ribose is a substrate for several related macrodomain proteins such as the human MacroD1, human MacroD2, *Escherichia coli* YmdB. In *E. coli*, YmdB is an RNase III inhibitor that modulates many different functions including biofilm formation (Kim et al., 2013), and *E. coli* adaptive resistance to aminoglycosides via an antibiotic stress-induced sequential modulation of the endoribonucleolytic activity of RNase III and RNase G (Song et al., 2014).

Regarding the microRNA-size small RNA fragments (micro*Ab*sRNA_4_ and its pre-micro*Ab*sRNA_4_ as well as the micro*Ab*sRNA_5_ and its pre-micro*Ab*sRNA_5_), our data suggest that these sRNAs exist in a premature form (pre-micro*Ab*sRNA) that act as precursors of their mature form, micro*Ab*sRNAs, likely obtained cutting the premature form. Moreover, the under-expressed micro*Ab*sRNA_4_ and its pre-micro*Ab*sRNA_4_ targeting a gene coding a structural membrane protein with a CD similar to cytoskeletal protein RodZ related to the cell-cycle control, cell division and chromosome partitioning, could speculatively address its regulation of these functions. In fact, the GO-term prediction highlighted two possible molecular functions, a DNA polymerase activity and a DNA-binding function. Furthermore, the micro*Ab*sRNA_4_ target (A1S_0501) was previously listed as a transcript that decreased significantly upon exposure to NaCl in *A. baumannii*, but no relationship with the mechanisms of antimicrobial tolerance in response to monovalent cations was previously found (Hood et al., 2010).

For the over-expressed micro*Ab*sRNA_5_ and its pre-micro*Ab*sRNA_5_, targeting a signal peptide involved in the cytokinin biosynthetic process, we have to keep in mind that *A. baumannii* is a versatile pathogen that can adhere and invade numerous cell types displaying varying degrees of susceptibility to invasion, stimulating the pro-inflammatory immune response (Choi et al., 2008). The stimuli and signaling pathways implicated in cell death are not yet established; however, they involve imbalanced calcium homeostasis, pro-inflammatory cytokines, and oxidative stress, all traits related to strain virulence (Smani et al., 2011; Mortensen and Skaar, 2012). Micro*Ab*sRNA_5_ can regulate host-microbe *A. baumannii* interactions that shape the pathogenesis of *Ab* infection mediated by the host immune response.

In this study, we experimentally and computationally discovered five statistically significant DE sRNAs characterizing COL^R^ *Ab* strains speculatively implicated, as *cis*-antisense sRNAs, in the regulation of protein synthesis machinery *via* under-expressed *Ab*sRNA_1_ and *Ab*sRNA_2_ and in different biological functions, including biofilm production, virulence and aminoglycoside-resistance *via* an under-expressed *Ab*sRNA_3_. Likewise, we found two micro*Ab*sRNAs that may be involved in cell-cycle control *via* an under-expressed micro*Ab*sRNA_4_ and in the host-microbe interaction *via* an over-expressed micro*Ab*sRNA_5_.

Colistin resistance onset in *A. baumannii* entails dissimilar biological adaptations – not exclusively related to colistin-resistance – supporting the extremely complex and dynamic nature of this life-threatening microorganism and the urgent need to elucidate the role of small RNAs, whose only the tip of the iceberg is known.

This work offers a model for the identification of sRNA signatures and the prediction of their targets in *A. baumannii*. Although we do not have clear information on their functions yet, our bioinformatic analysis may provide indications regarding the cellular roles of these new sRNAs.

## Data Availability Statement

The datasets generated for this study can be found in the NCBI GEO Database: GSE109951.

## Author Contributions

VC and SSte conceived and designed the study. VC, SStr, FL, VD, and AZ performed the genomics, transcriptomics, real time qPCR, and bioinformatics. GP contributed to the bioinformatics analysis. JA carried out the mutant construction. All authors analyzed the data and contributed and approved the manuscript.

## Conflict of Interest

The authors declare that the research was conducted in the absence of any commercial or financial relationships that could be construed as a potential conflict of interest.
