# Supplementary material for: COLR Acinetobacter baumannii sRNA Signatures: Computational Comparative Identification and Biological Targets
Source: Front Microbiol. 2020 Jan 17;10:3075. doi: 10.3389/fmicb.2019.03075 (PMC6978653; doi:10.3389/fmicb.2019.03075)
Supplement: Supplementary file 1 [file Data_Sheet_1.PDF]

Figure S1. Growth curve

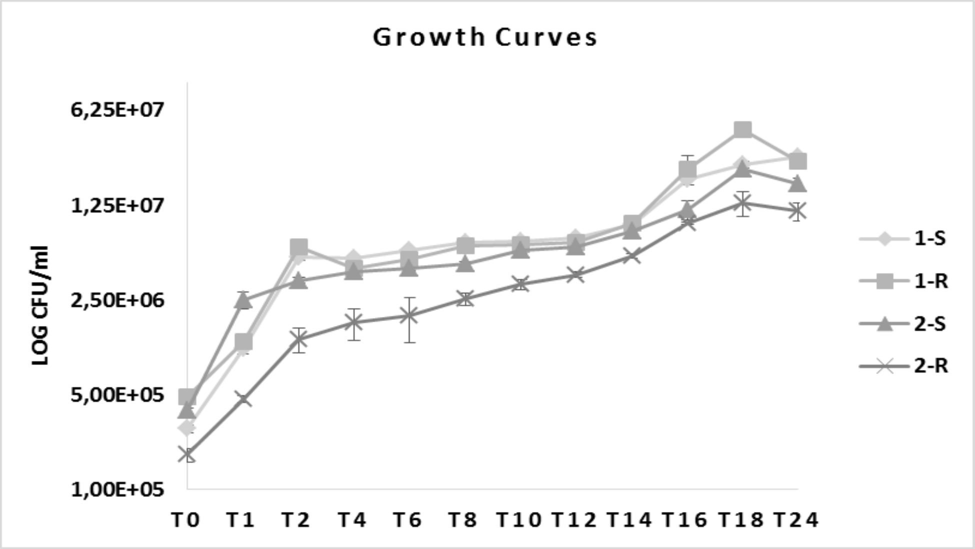

Showed values are the means of three replicates with standard deviation.
1-S, 2-S COL^S *A. baumannii*
1-R, 2-R COL^R *A. baumannii*
