# Supplementary material for: COLR Acinetobacter baumannii sRNA Signatures: Computational Comparative Identification and Biological Targets
Source: Front Microbiol. 2020 Jan 17;10:3075. doi: 10.3389/fmicb.2019.03075 (PMC6978653; doi:10.3389/fmicb.2019.03075)
Supplement: Supplementary file 2 [file Data_Sheet_2.PDF]

Table S1. Rockhopper data analysis of selected sRNAs

a) Strain-pair 1

sRNA start	sRNA stop	Strand	Name	RPKM* 1-R	RPKM* 1-S	p-value	*q*-value
193854	193835	-	antisense: A1S_r01	175	100	0,631821	1
194025	193996	-	antisense: A1S_r01	510	116	0,001676	0,035688
194124	194088	-	antisense: A1S_r01	177	144	0,61656	1
194430	194283	-	antisense: A1S_r01	110	174	0,136487	0,536446
194580	194546	-	antisense: A1S_r01	105	523	8,31E-05	0,002943
194823	194802	-	antisense: A1S_r01	173	636	0,005432	0,085957
2904500	2904426	-	antisense: A1S_2505	187	647	0,000289	0,008645
2904950	2904920	-	antisense: A1S_2505	374	276	0,274232	0,728338
2944392	2944318	-	antisense: ACICU_02783	181	630	7,50E-05	0,004447
2944842	2944812	-	antisense: ACICU_02783	362	269	0,403229	0,8789
545842	545822	-	antisense: A1S_0501	111	696	0,000138	0,004463
564585	564555	-	antisense: ACICU_00510	93	506	2,20E-05	0,001583
3577374	3577394	+	antisense: A1S_3097	173	49	0,771877	1
3577445	3577464	+	antisense: A1S_3097	173	0	0	0
3498176	3498196	+	antisense: ACICU_03297	168	48	0,778366	1
3498247	3498266	+	antisense: ACICU_03297	168	0	1	1

* RPKM = Reads per kilo base per million mapped reads

b) Strain-pair 2

sRNA start	sRNA stop	Strand	Name	RPKM* 2-R	RPKM* 2-S	p-value	*q*-value
194033	193995	-	antisense: A1S_r01	19	780	1,68E-09	1,55E-07
194107	194088	-	antisense: A1S_r01	0	218	1	1
194432	194327	-	antisense: A1S_r01	38	321	0,022142	0,574301
194580	194548	-	antisense: A1S_r01	204	875	0,078538	1
194823	194802	-	antisense: A1S_r01	97	343	0,796859	1
2904495	2904426	-	antisense: A1S_2505	6	352	1,9E-08	1,61E-06
2904941	2904924	-	antisense: A1S_2505	95	171	0,660129	1
2944387	2944318	-	antisense: ACICU_02783	6	347	1,12E-08	1,31E-06
2944833	2944816	-	antisense: ACICU_02783	94	169	0,649392	1
546023	545917	-	antisense: A1S_0501	5	310	1,38E-07	5,92E-06
545861	545823	-	antisense: A1S_0501	16	257	0,214477	1
564594	564556	-	antisense: ACICU_00510	16	253	0,930366	1
3577336	3577563	+	antisense: A1S_3097	472	15	7,89E-50	1,51E-47
3498138	3498365	+	antisense: ACICU_03297	466	15	2,83E-34	7,59E-32

* RPKM = Reads per kilo base per million mapped reads
